# Phlorotannins from *Undaria pinnatifida* Sporophyll: Extraction, Antioxidant, and Anti-Inflammatory Activities

**DOI:** 10.3390/md17080434

**Published:** 2019-07-24

**Authors:** Xiufang Dong, Ying Bai, Zhe Xu, Yixin Shi, Yihan Sun, Srinivas Janaswamy, Chenxu Yu, Hang Qi

**Affiliations:** 1 School of Food Science and Technology, Dalian Polytechnic University, National Engineering Research Center of Seafood, Dalian 116034, China; 2 Department of Dairy and Food Science, South Dakota State University, Brookings, SD 57007, USA; 3 Department of Agricultural and Biosystems Engineering, Iowa State University, Ames, IA 50011, USA

**Keywords:** *Undaria pinnatifida* sporophyll, phlorotannin extracts, prepared processing, antioxidant activity, anti-inflammatory activity

## Abstract

*Undaria pinnatifida* sporophyll (*U. pinnatifida*) is a major byproduct of *U. pinnatifida* (a brown algae) processing. Its phenolic constituents, phlorotannins, are of special interest due to their intrinsic ability to precipitate proteins. Herein, a high-temperature extraction procedure was used to isolate these biologically active compounds. The heating temperature, heating time, and extraction solvent (ethanol) concentration were examined with response surface analysis to determine the optimal conditions to achieve the maximum extraction rate. The analysis revealed the optimal conditions to be: 170 °C of heating temperature, 5.2 h of heating time, and 52% ethanol concentration for a yield of 10.7 ± 0.2 mg gallic acid equivalent/g dry weight (GAE/g DW) of sample. Compared to epigallocatechin gallate (EGCG), the extracted phlorotannins displayed higher antioxidant activity on H_2_O_2_-induced RAW 264.7 cells with improved efficiency. Furthermore, the compounds exhibited strong anti-inflammatory activity. These findings potentially can be utilized to guide development of novel functional foods and food supplements from sea-originated resources such as brown algae.

## 1. Introduction

Polyphenolics are natural bioactive compounds commonly found in fruits, vegetables, and cereal grains. They are best known for their health beneficial properties and have been attracting considerable attention among food processors and nutritionists [1]. The burgeoning field of substituting synthetic compounds by natural bioactive ingredients in food, pharmaceutical, and nutraceutical applications requires research on natural materials [2,3]. An emerging trend is to extract bioactive compounds such as polyphenolics from inexpensive and abundant sources [4,5], among which seaweeds stand out as promising candidates.

Seaweeds have long been part of human diets and medicine [6]. They are among major sources of industrially important metabolites such as polysaccharides and polyphenols [7]. In addition, they also contain a wide range of bioactive secondary metabolites such as phenolic compounds, terpenoids, and nitrogen compounds. Among the seaweeds, algae, composed of a highly heterogeneous group of organisms, are gaining attraction as functional food ingredients [8]. In this family, brown algae (*Phaeophyceae*) are well-known for possessing phenolic compounds [9] with antioxidant [10,11], anti-inflammatory [12], antidiabetic [13], anti-proliferative [14], and antibacterial [10] activities. Among the long list of phenolic compounds, phlorotannins are of special interest because of their intrinsic ability to precipitate proteins. Phlorotannins could be utilized as inhibitors of glucosidase and amylase as well as to delay absorption of dietary carbohydrates [15,16]. They exhibit a high protective effect on fish mince for quality enhancement, especially in terms of controlling lipid oxidation and Ca^2+^-ATPase [17].

Phlorotannins are generally found as complexes within algae cell wall components [18], which greatly limits its extraction with common protocols used typically for fruits, vegetables, and grains which have less rigid cell wall structures. Hence, utilization of advanced extraction procedures such as centrifugal partition extraction (CPE), supercritical fluid extraction (SFE), pressurized liquid extraction (PLE), and enzyme-assisted extraction (EAE) [18] has become necessary. One such processes is high-temperature extraction. In this method, heat leads to hydrolysis of cross-linked cell wall structures and facilitates the release of heat-resistant bioactive components (e.g., small molecular weight compounds) to increase the overall extraction efficiency. To optimize the operation, different heating temperatures coupled with various solvent concentrations and heating times need to be explored.

*Undaria pinnatifida* (*U. Pinnatifida*) is a brown alga that normally grows in the seas of China, Korea, and Japan. During 2015, around 193,000 tons were harvested in China [19], while the worldwide production reached 2,359,000 tons (FAO, 2016). The structure of *U. pinnatifida* can be categorized into blade (lamina), midrib, sporophyll, and root-like formations (haptera). It is also known as wakame, the name of the processed *U. pinnatifida* [20]. Sporophyll, the reproductive organ of *U. pinnatifida*, is a processing by-product of the seaweed food industry. It is rich in protein and bioactive compounds; therefore, it is a valuable raw material for further processing to yield functional supplements and products [21]. Sporophyll is rigid, and its tissue structure is compact and dense. To effectively process it, relatively high temperature heating is needed.

Herein, in this study, a high-temperature extracting procedure was developed to isolate phlorotannin extracts from *U. pinnatifida* sporophyll (PEUPS). The heating temperature, heating time, and extraction solvent (i.e., ethanol) concentration were studied by the response surface method (RSM) to determine the optimal conditions to achieve the maximum extraction rate. In addition, PEUPS were evaluated for their antioxidant and anti-inflammatory activities.

## 2. Results and Discussion

### 2.1. Single-Factor Experiment

#### 2.1.1. Effect of Ethanol Concentration on Total Phlorotannin Content (TPC) and Antioxidant Activity of the Phlorotannin Extracts from U. pinnatifida Sporophyll (PEUPS)

**Figure 1:**
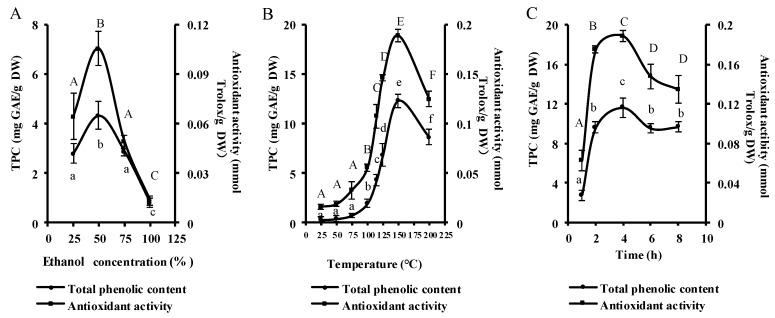
The extraction characteristics of phlorotannin extracts from *U. pinnatifida* sporophyll (PEUPS) extracted under different extraction conditions. The total phenolic content (TPC) and antioxidants were determined by changing the ethanol percentage in ethanol concentration (**A**), temperature (**B**), and time (**C**). The results were expressed as mg GAE/g DW and mmol trolox/g DW, respectively.

In order to assess the effect of ethanol concentration on the total phlorotannin content (TPC) and antioxidant activity of the PEUPS, four ethanol concentrations (25%, 50%, 75%, and 100%) were tested with heating temperature of 115 °C and heating time of 4 h. As shown in Figure 1A, the TPC increased from 2.78 ± 0.40 to 4.33 ± 0.57 mg Gallic acid equivalent/g dry weight (GAE/g DW) when ethanol went from 25% to 50%, but it subsequently decreased to 2.88 ± 0.19 and finally to 0.80 ± 0.18 mg GAE/g DW at 75% and 100% ethanol, respectively. A similar trend was noticed in the antioxidant activity of the PEUPS; it rose from 0.06 ± 0.01 to 0.11 ± 0.01 mmol Trolox/g DW as ethanol went from 25% to 50%, but then it dropped to 0.05 ± 0.004 and 0.01 ± 0.01 mmol Trolox/g DW at 75% and 100% ethanol, respectively. It appeared that the ethanol concentration of 50% was the most appropriate for obtaining the highest amounts of TPC and the highest antioxidant activity. This phenomenon might be due to the solubility differences among phenolic chemicals in solvents with different polarities. To extract the most TPC, a mixed system with a solvent of different polarity performs better than a single solvent. These results agreed very well with the phlorotannin extraction from *Eisenia Bicyclis* [18], perennial brown algae from the *Laminariaceae* family that forms an integral part of traditional Japanese cuisine.

#### 2.1.2. Effect of Extraction Temperature on TPC and Antioxidant Activity of the PEUPS

The TPC and antioxidant activity of PEUPS were monitored at eight extraction temperatures (25, 50, 75, 100, 115, 125, 150, and 200 °C) with 50% ethanol for 4 h. The TPC surged steadily from 0.30 ± 0.30 GAE/g DW at 25 °C to 12.30 ± 0.66 mg GAE/g DW at 150 °C, and it later dropped to 8.62 ± 0.80 mg GAE/g DW at 200 °C (Figure 1B). Similarly, the antioxidant activity rose from 0.02 ± 0.002 mmol Trolox/g DW at 25 °C to 0.19 ± 0.01 mmol Trolox/g DW at 150 °C and settled at 0.12 ± 0.01 mmol Trolox/g DW at 200 °C. Thus, it appeared that 150 °C could be the optimal temperature for maximum extraction of phlorotannin from *U. pinnatifida* sporophyll. In the case of *Eisenia bicyclis*, a gain in yield was noticed with a temperature surge from 20 to 80 °C [18], but further increase of temperature did not enhance the yield, possibly due to heat-induced degradation of some of the phlorotannins. In comparison, previous studies on extraction from the *Sargassum muticum* [22,23], an invasive brown macroalga widely spread across the European Atlantic coast, suggested 150 °C was the optimal temperature. In general, proper heat treatment could enhance the solubility of phlorotannins, it can also disrupt the cell wall structure of plant cells and soften plant tissues to ease the release of phlorotannins to facilitate their extraction. However, excessive heating seems not to further increase the extraction yield.

#### 2.1.3. Effect of Extraction Time on TPC and Antioxidant Activity of the PEUPS

In order to investigate the effect of extraction time on the TPC and antioxidant activity of the PEUPS, heating times of 1, 2, 4, 6, and 8 h in the condition of 150 °C and 50% ethanol were used. Figure 1C shows an increasing trend for both TPC and the antioxidant activity from 1 to 4 h. The TPC increased from 2.77 ± 0.51 to 11.64 ± 0.99 mg GAE/g DW, and antioxidant activity enhanced from 0.06 ± 0.01 to 0.18 ± 0.01 mmol Trolox/g DW. Subsequent increase of heating time to 8 h resulted in decreased TPC to 9.65 ± 0.52 mg GAE/g DW and antioxidant activity to 0.13 ± 0.01 mmol Trolox/g DW, respectively. Thus, it appeared that 4 h of heating time was optimal. This observation agrees well with phlorotannin extraction from the seaweed *Sargassum muticum* [22]. Excessive heating seems to have a negative impact on the extraction of PEUPS, most likely due to heat-induced degradation of some of phlorotannins.

### 2.2. Response Surface Method (RSM) Model

To optimize the controlling parameters of the PEUPS extraction, the independent variables (extraction time, ethanol concentration, temperature) were analyzed by RSM (Table 1). The PEUPS were extracted with the 17 selected combinations of the controlling parameters, as shown in Table 2. Table 3 presents the predicted TPCs by the RSM model. ANOVA was used to examine the statistical significance of the model. The *p*-value Prob > F for the regression model of TPC was 0.0399, indicating that the model was a reasonably good fit to the experimental data. The experimental values for the TPC matched well with the predicted values, indicating a satisfactory model (Table 4).

**Figure 2:**
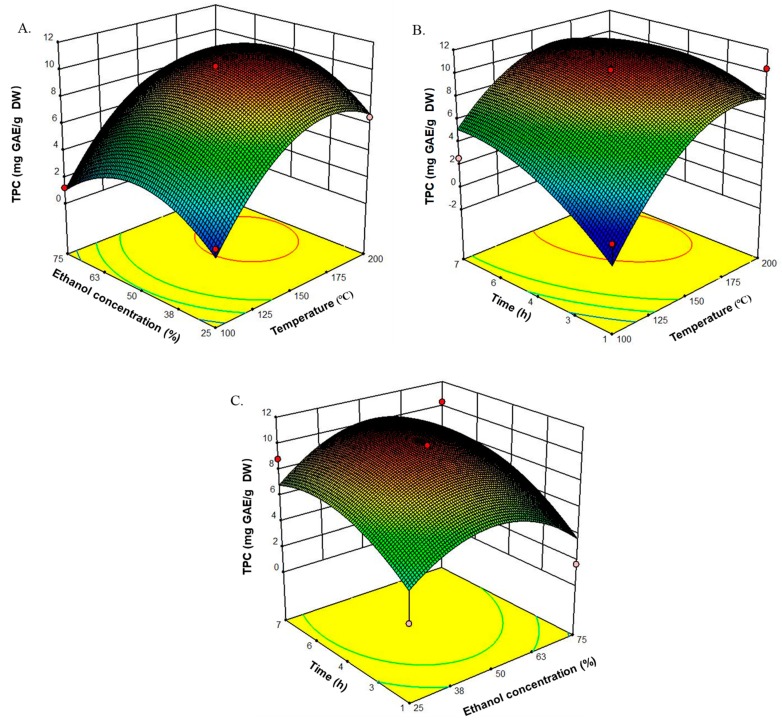
Standardized Box–Behnken design for the three response variables studied in the experimental design and their corresponding response surfaces. (**A**) Effects of ethanol concentration (25%, 50%, and 75%) and temperature (100, 150, and 200 °C) on TPC. (**B**) Effects of time (1, 4, and 7 h) and temperature (100, 150, and 200 °C) on TPC. (**C**) Effects of time (1, 4, and 7 h) and ethanol concentration (25%, 50%, and 75%) on TPC.

The mean experimental TPC varied from 1.2 to 10.3 mg GAE/g DW. Using the RSM model mentioned above, three-dimensional (3D) response surface plots were obtained to spot the region of optimal TPC extraction (Figure 2). Temperature appeared to have the greatest influence on TPC of the PEUPS. On the other hand, a subtle, significant, negative quadratic effect of extraction time (*p* < 0.1) for the TPC extraction was noticed, indicating that there was a maximum yield point in the TPC at an extraction time of 5.2 h (Figure 2B,C). TPC yield started to decrease at higher extraction time. Further increase in the extraction time may lead to degradation of the phlorotannins through heat-induced chemical degradation or reaction with other cell components [24]. The ethanol concentration was another important parameter in the extraction procedure. As shown in Figure 2A,C, the TPC increased with ethanol concentration up to 52%, followed by gradual reduction with a minimum at 100%. A remarkable drop in the TPC at 100% ethanol concentration revealed the low phlorotannin solubility in pure ethanol. This observation indicated that the extraction of phlorotannins largely depended on the polarity of the solvent. Thus, it appeared that one single solvent might not be effective to isolate the total bioactive compounds. The extraction temperature also appeared to be significant. As shown in Figure 2A,B, the linear and quadratic interactions with time were significant on the TPC. With a rise in the temperature, the TPC adopted a linear effect for temperatures less than 170 °C, indicating the role of temperature on the overall yield of TPC. Overall, the optimal conditions obtained were extraction temperature of 169.2 °C, time of 5.2 h, and ethanol concentration of 52%, with a predicted 11.0 mg GAE/g DW TPC. These values correlated well with the experimental values of TPC 10.7 ± 0.2 mg GAE/g DW at a temperature of 170 °C with an extraction time of 5.2 h using 52% ethanol. These conditions were used for further extraction of the PEUPS. Based on this optimal condition, the extracted PEUPS were analyzed using an Ultra-high performance liquid chromatography-triple four-stage bar-time-of-flight/mass spectrometry (UPLC-Triple-TOF/MS) system (Acquity^TM^ ultra, Waters, USA). There were nine small molecular weight polyphenolic compounds identified including 3,5-dihydroxyphenol, 3,4-dihydroxybenzaldehyde, 5-acetoxy-2-hydroxybenzaldehyde, hydroquinone, hydroquinone monoacetate, 4-hydroxy-benzaldehyde, C_8_H_8_O_3_, 1-(3-methoxy-4-hydroxyphenyl) ethenone, and 4-methoxy-phenol (as shown in supplementary Figure S1). These small molecular weight phenolic compounds were thermal stable. Although high-temperature extraction tends to become less effective due to possible heat-induced chemical degradation when the heating becomes excessive, as shown by our data, it appeared to be an efficient way of harvesting small molecular weight phenolic compounds, which, as shown below, are also quite valuable as antioxidant and anti-inflammatory agents.

### 2.3. Antioxidant Activity of the PEUPS Evaluated with RAW 264.7 Macrophages

Before the activity analysis, the PEUPS at six selected concentrations of 2.5, 5, 10, 20, 40, and 80 μg/mL were cultured with cells for 24 h to demonstrate that PEUPS do not exert any toxic effect. We found the RAW 264.7 macrophages viability was not significantly (*p* > 0.05) influenced except for the 80 μg/mL concentration. However, the cell viability was much higher than 80% (as shown in supplementary Figure S2). To investigate the antioxidant and anti-inflammatory properties of the PEUPS, we used RAW 264.7 macrophage cells as a model system and epigallocatechin gallate (EGCG) as a positive control. We designed and conducted our experiments (3.8 and 3.9) for two scenarios: (1) “post-stimuli (procedure 1)”, where the PEUPS/EGCG were administered to the cells after the stimuli (H_2_O_2_ or lipopolysaccharide (LPS)) was introduced, was used to mimic a therapeutic treatment scenario in which the agents were used to repair damages already caused by the stimuli; and (2) “pre-stimuli (procedure 2)”, where the PEUPS/EGCG were administered to the cells before the stimuli (H_2_O_2_ or LPS) was introduced, was used to mimic a preventive treatment scenario in which the agents were used to protect the cells from damages to be caused by the stimuli. Both scenarios are meaningful in terms of evaluating the bioactivities of the PEUPS.

**Figure 3:**
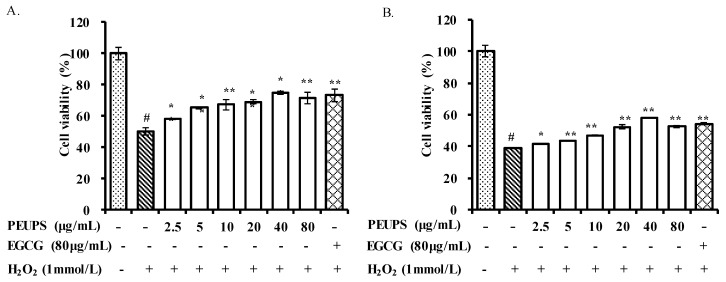
Effects of PEUPS on cell viability in H_2_O_2_-induced RAW 264.7 cells. (**A**) Effect of PEUPS on cell viability in H_2_O_2_-induced RAW 264.7 cells; the cells were treated using procedure 1. (**B**) Effect of PEUPS on cell viability in H_2_O_2_-induced RAW 264.7 cells; the cells were treated using procedure 2. Cells (4 × 10^5^ cells/mL) were indicated (2.5, 5, 10, 20, 40, and 80 μg/mL) of PEUPS and 1 mmol/L of H_2_O_2_. The cell viability assay was performed using methyl thiazolyl tetrazolium (MTT) assay. Values are means ± SE (*n* = 3). # *p* < 0.01 compared with control group; * *p* < 0.05, ** *p* < 0.01 compared with H_2_O_2_ group alone.

As shown Figure 3A, under the post-stimuli scenario, the viability of RAW 264.7 cells (blank) decreased markedly (*p* < 0.05) upon exposure to 1 mmol/L of H_2_O_2_. After treatment with different concentrations of the PEUPS (2.5–80 μg/mL), or EGCG at a concentration of 80 μg/mL (positive control), the viability of the RAW 264.7 cells improved. Approximately, the cell viability at 40 μg/mL PEUPS treatment was already at the same level of the positive control. For the pre-stimuli scenario, as shown Figure 3B, the viability in the RAW 264.7 cells also decreased markedly (*p* < 0.05), and it was much lower than that in Figure 3A. Different concentrations of PEUPS (2.5–80 μg/mL) could protect the cells to different degrees. EGCG at a concentration of 80 μg/mL also had approximately the same cell viability as the PEUPS at 80 μg/mL. However, overall, the preventive mode worked less well than the therapeutic mode (Figure 3A), suggesting these agents were better at repair than at prevention. Reactive oxygen species (ROS) are key mediators for cell death. Elimination of stimulation-induced ROS through the use of antioxidants such as polyphenol could protect cells from those stimuli [25]. Hence, it is reasoned that the antioxidant activities of the PEUPS play key roles in reducing cell deaths. Our results were consistent with previous studies, which reported that phlorotannins can be developed into a potential bio-molecular candidate to inhibit ROS formation [26].

### 2.4. Anti-Inflammatory Activity of PEUPS in the RAW 264.7 Macrophages

**Figure 4:**
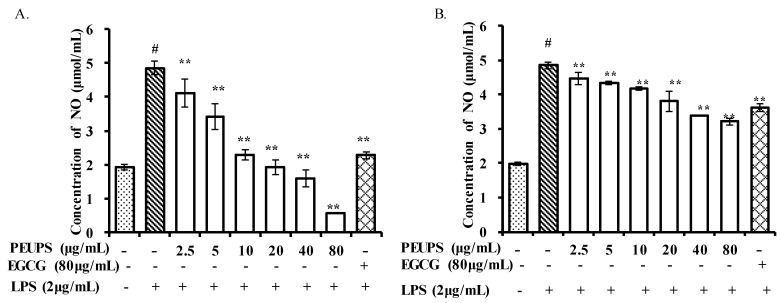
Effects of PEUPS on NO production in lipopolysaccharide (LPS)-stimulated RAW 264.7 cells. (**A**) Effect of PEUPS on cell viability; the cells were treated using procedure 1. (**B**) Effect of PEUPS on NO production; the cells were treated using procedure 2. LPS was the control and epigallocatechin gallate (EGCG) was the positive control. Cells (4 × 10^5^ cells/mL) were indicated concentrations (2.5, 5, 10, 20, 40, and 80 μg/mL) of PEUPS and 2 μg/mL of LPS, respectively. The nitrite concentration in the medium was determined using Griess reagent. Values are means ± SE (*n* = 3). # *p* < 0.01 compared with control group; * *p* < 0.05, ** *p* < 0.01 were compared with the control group.

The chronic presence of nitric oxide (NO) is associated with various carcinomas and inflammatory conditions [27]. The anti-inflammatory characteristics of phlorofucofuroeckol A isolated from *Ecklonia cava* has been studied in LPS-stimulated RAW 264.7 cells [28]. Since NO produced by inducible nitricoxide synthase (iNOS) is one of the inflammatory mediators, the effects of various PEUPS concentrations on NO production in LPS-activated RAW 264.7 cells were evaluated in vitro. As shown in Figure 4, lipopolysaccharide (LPS) stimulation resulted in a significant NO hike in the cell culture. However, treating affected cells with PEUPS (post-stimuli scenario) decreased the production of NO at all tested concentrations (Figure 4). In addition, as confirmed by the immunomodulatory activity assessment, the PEUPS did not show any cytotoxic effect on the RAW 264.7 cells. The NO production of PEUPS at the concentration of 80 μg/mL (procedure 1) was 0.57 ± 0.02 μmol/mL. Cells treated with EGCG at a concentration of 80 μg/mL had approximately the same NO production as those treated with the PEUPS at 10 μg/mL. The NO production decreased significantly, and this trend agreed well with the findings reported from Kim et al. [27] on the effect of phlorofucofuroeckol A, dieckol, and dioxinodehydroeckol on NO production in the LPS-induced RAW 264.7 cells: phlorofucofuroeckol A was found to significantly reduce LPS-induced NO production in a dose-dependent manner at the concentration of 5–20 µM [29]. As for preventive effects shown in Figure 4B, the trend of NO production under scenario “post-stimuli” was similar to Figure 4A: the higher the exposure to the PEUPS, the less NO production in the cells; hence, a larger anti-inflammatory effect can be achieved. The NO production with PEUPS treatment at the concentration of 80 µg/mL was 3.21 ± 0.11 µmol/mL, and it appeared that the PEUPS was a better anti-inflammatory agent than EGCG at the same concentration. The PEUPS can be considered as a potential agent for suppressing the NO production without any cytotoxic effect, both as a therapeutic agent and as a preventive agent, though the preventive effect is less significant.

**Figure 5:**
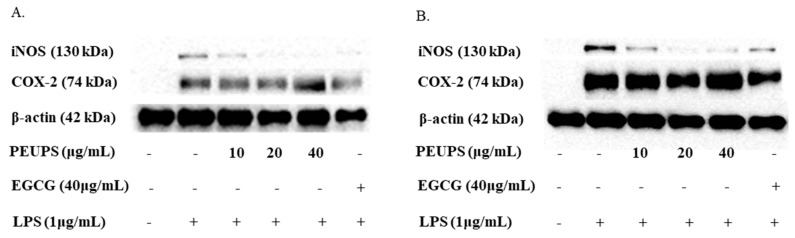
Effect of PEUPS on LPS-induced iNOS and COX-2 protein expression in RAW 264.7 macrophages. (**A**) The cells were treated using procedure 1. (**B**) The cells were treated using procedure 2. Then, cell lysates were electrophoresed, and the expression levels of iNOS and COX-2 were detected with specific antibodies.

In order to further characterize the inhibitory effect of the PEUPS on the NO production, a western blot analysis was carried out to evaluate the expression of key proteins/enzymes (iNOS and COX-2) in the cells. iNOS is a catalytic enzyme to produce NO, and cyclooxygenase-2 (COX-2) is one of the inducible enzymes in excessive inflammatory responses that can regulate the production of prostaglandins. iNOS and COX-2 gene expressions are primarily regulated at the transcriptional level, and their inductions are largely dependent on activities of transcription factors that interact with respective cognate cis-acting elements within the iNOS or COX-2 promoters [27,30,31]. iNOS and COX-2 protein expressions were markedly increased when the RAW 264.7 cells were treated with LPS compared to the control without LPS (Figure 5). As shown in Figure 5A, the iNOS protein expressions were significantly inhibited by the PEUPS in a dose-dependent manner. Moreover, the iNOS protein expression was almost completely suppressed at a concentration of 40 μg/mL of PEUPS. As shown in Figure 5B, pretreatment with the PEUPS (10, 20, and 40 μg/mL) predominantly inhibited iNOS protein expression, and iNOS protein expressions were almost completely suppressed at both 20 and 40 μg/mL of PEUPS. All these results clearly pointed out that the PEUPS reduce the production of NO mainly by down-regulating iNOS protein expression. It seemed not to inhibit COX-2 protein expression (Figure 5A,B) much, different from EGCG, which down-regulates both iNOS and COX-2. Nonetheless, PEUPS is clearly a good anti-inflammatory agent that could reduce stimulated NO production in RAW 264.7 macrophages.

## 3. Materials and Methods

### 3.1. Samples and Chemicals

*U. pinnatifida* sporophyll was obtained from Dalian Aquatic Culture Group Company of Liaoning province (China) in October 2014. Gallic acid, phloroglucinoldihydrate, lipopolysaccharide (LPS), and 2,2′-azino-bis(3-ethylbenzothiazoline-6-sulfonic acid (ABTS, P99%) were purchased from Sigma-Aldrich (St. Louis, MO, USA). The Folin–Ciocalteu phenol reagent was provided by Merck (Shanghai, China). Dulbecco’s modified Eagle’s medium (DMEM) and penicillin-streptomycin solution were obtained from HyClone (Logan, UT, USA). Fetal bovine serum (FBS) was obtained from Sangon Biotech Co., Ltd. (Shanghai, China). Dimethyl sulfoxide (DMSO) was obtained from Yeasen Biotech Co., Ltd. (Shanghai, China). NO was obtained from Jiancheng Institute of Biotechnology (Nanjing, China). Anti-mouse iNOS, anti-mouse COX-2, anti-β-actin rabbit polyclonal antibody, anti-rabbit IgG, and horseradish peroxidase (HRP)-linked antibody were purchased from Cell Signaling Technology, BCG, Boston, MA, USA. Other reagents were obtained from Sangon Biotech Co., Ltd. (Shanghai, China).

### 3.2. Sample Preparation

*U. pinnatifida* sporophyll was dried in an oven at 80 °C for 48 h. Then it was put into a dusting machine and sifted through a 40-mesh sieve. The powder was rehydrated for 4 h at a mass:water ratio of 1:10, centrifuged at 10,000× *g*, 4 °C for 10 min, and the pallet was collected and stored at −4 °C for further use.

### 3.3. Extraction of PEUPS

Extractions were carried out using an accelerated solvent extractor that was equipped with a solvent controller unit. Ultrapure water and ethanol were used as solvents. Initially, solvents were sonicated for 10 min. For each experiment, 1 g of the rehydrated powder of *U. pinnatifida* sporophyll was loaded into a 50 mL stainless steel extraction cell with 10 mL solvent. The experiments were carried out by varying the static extraction time (1, 2, 4, 6, and 8 h), extraction temperature (25, 50, 75, 100, 115, 125, 150, and 200 °C), and percentage of ethanol in the extraction solvent mixture (25%, 50%, 75%, and 100%). After treatment at different conditions, ethanol was removed using a rotary evaporator, and phlorotannin was reconstituted with water. The PEUPS were then kept at 4 °C before use within 24 h.

### 3.4. Experimental Design

The extraction was optimized using a three-level factorial design (including two center points). The effects of temperature (100, 150, and 200 °C), percentage of ethanol (25%, 50%, and 75%), and heating time (1, 4, and 7 h) on TPC (mg GAE/g DW) were investigated. A total of 17 experiments were conducted in a randomized order. Design and data analysis were carried out using response surface method with Design-Expert 8.0.5. The effects of the independent variables on these response variables in the extraction process were assessed using the pure error, considering a level of confidence of 95% for all the variables. The quadratic model proposed for each response variable (Y) was:

Y = β0 + β1 × A + β2 × B + β3 × C + β12 × A × B + β13 × A × C + β23 × B × C + β11 × A2 + β22 × B2 + β33 × C2,(1)

where A is the solvent composition (percentage of ethanol in the mixture); B is the temperature; C is the heating time; β0 is the intercept; β1, β2, and β3 are the linear coefficients; β11, β22, and β33 are the quadratic coefficients; and β12, β13, and β23 are the linear-by-linear interaction coefficients. The significant criteria for each polynomial model were based on the correlation coefficients (R2), RSM, and the lack-of-fit tests from the analysis of variance table. The effect of each factor and its statistical significance were analyzed from the standardized Pareto chart. The response surfaces of the respective mathematical models were also obtained, and the significances were accepted at *p* < 0.05 (level of confidence of 95%). A multiple-response optimization was carried out by the combination of experimental factors and maximizing the desirability function.

### 3.5. TPC Measurement (Folin–Ciocalteu Method)

TPC was determined spectrophotometrically by using the Folin–Ciocalteu method, with some modifications. Briefly, 50 μL of extract solution and 600 μL of ultrapure water were mixed, and 50 μL of undiluted Folin–Ciocalteu reagent was added. After 1 min, 150 μL of 20% (w/v) Na_2_CO_3_ was added, and the volume was made to 1 mL with water. The samples were incubated for 2 h at 25 °C in the dark. Later, 200 μL of reaction mixture was transferred to a 96-well microplate. The absorbance was measured at 760 nm by Microplate Reader (Tecan Infinite, Switzerland, M200). Standard curves with serial gallic acid solutions were generated for calibration. The phenolic content was expressed as milligrams of gallic acid equivalent per gram of dried weight (mg GAE/g DW) of sample. All analyses were done in triplicate and average values reported.

### 3.6. Trolox Equivalents Antioxidant Capacity Assay (TEAC)

Trolox equivalents antioxidant capacity (TEAC) assay was employed to measure the antioxidant capacity. ABTS^+^ radical was produced by reacting 7 mM ABTS and 2.45 mM potassium persulfate in the dark at room temperature for 16–24 h before use. The aqueous ABTS^+^ solution was diluted with 200 mM phosphate buffer (pH 7.4) to an absorbance of 0.7 ± 0.02 at 734 nm. Ten microliters of sample (5 different concentrations ranging from 0.25 to 2 mg/mL) and 1 mL of ABTS^+^ solution were mixed in an Eppendorf vial, and 200 µL of the mixture was transferred into a 96-well microplate. The absorbance was measured at 734 nm every 5 min for 45 min in a M200 Microplate Reader (Tecan Infinite, Switzerland). Trolox was used as reference, and results were expressed as TEAC values (mmol of trolox/g the dried weight of sample). The values were obtained from five different concentrations of each tested in the assay giving a linear response between 20% and 80% of the blank absorbance. All analyses were done in triplicate and average values reported.

### 3.7. Cell Culture

The macrophage-like cell line, RAW 264.7, has previously been used to characterize the immunomodulatory action of various components at the molecular level [18,22]. RAW 264.7 cell line was obtained from Shanghai Institute of Cell Biology (Shanghai, China) and maintained in DMEM supplemented with heat-inactivated 10% fetal bovine serum, 100 U/mL penicillin, and 100 U/mL streptomycin in a humidified atmosphere of 5% CO_2_ at 37 °C. When cells reached sub-confluence, they were harvested with trypsin-EDTA and diluted to a suspension in a fresh medium. The cells were seeded in 96-well plates with 4 × 10^5^ cells/mL and allowed to adhere for 24 h at 37 °C in a humidified atmosphere containing 5% CO_2_.

### 3.8. Antioxidant Activity Analysis

Cells were treated following two protocols: Procedure 1 (post-stimuli type), the number of cells from the pretreated wells was quantified, then the growth medium was replaced with 100 µL medium containing 1 mmol/L H_2_O_2_ for 1 h. Then, the medium containing H_2_O_2_ was sucked out, and the cells were washed with HBSS (Hank’s balanced salt solution) twice, and the growth mediums containing different concentration of PEUPS (2.5, 5, 10, 20, 40, and 80 μg/mL) were added to the cells and cultured for 3 h. Procedure 2 (pre-stimuli), the number of cells from the pretreated wells was quantified, and the growth medium was replaced with 100 µL medium containing different concentrations of PEUPS (2.5, 5, 10, 20, 40, and 80 μg/mL) for 23 h. Then, the medium containing PEUPS was sucked out, the cells were washed with HBSS twice, and the growth medium containing 1 mmol/L H_2_O_2_ was added to the cells and cultured for 1 h. After treatment under each procedure, the cells were washed with HBSS twice and measured by a methyl thiazolyl tetrazolium (MTT) assay to evaluate their viability. Absorption values were recorded at 540 nm using M200 Microplate Reader (Tecan Infinite, Switzerland). The viability of RAW 264.7 cells in each well was calculated as the percentage of live cells. All analyses were done in triplicate and average values reported.

### 3.9. Determination of Nitric Oxide Production

Cells were treated the same as in Section 3.8 with pre-stimuli and post-stimuli procedures with 1 mmol/L H_2_O_2_ replaced by 2 µg/mL LPS. NO production was assessed indirectly by the accumulated nitrite in the culture supernatant, which is the stable end product of NO reacted with oxygen. Supernatant from each well was evaluated for NO with a commercial kit. All measurements were conducted in triplicate. The optical densities of the samples were detected at 540 nm. The NO production of RAW 264.7 cells in each well was calculated as the percentage of NO production with respect to nontreated LPS-stimulated RAW 264.7 cells. All analyses were done in triplicate and average values reported.

### 3.10. Western Blot Analysis

After cells were treated following Section 3.9, the RAW 264.7 cells were washed twice with ice-cold PBS and lysed with a buffer (50 mM Tris-HCl, pH 7.5, 150 mM NaCl, 1% Triton X-100, 1% Tween-20, 0.1% SDS, 10 g/mL leupeptin, 50 mM NaF, and 1mM phenylmethylsulfonyl fluoride) on ice for 1 h. After centrifugation at 14,000× *g* for 20 min, the protein content of supernatant was measured, and aliquots (20 μg) of protein solution were subject to sodium dodecyl sulfate-polyacrylamide gel electrophoresis (SDS-PAGE), and the separated proteins were transferred onto a polyvinylidene fluoride membrane. The membrane was blocked with 5% bovine serum albumin (BSA) in Tris-buffered saline Tween-20 (TBS-T) buffer for 1 h and incubated for 2 h with primary antibody (iNOS = 1:1000 dilution, monoclonal mouse anti-iNOS antibody, No. 2982S; COX-2 = 1:1000 dilution, anti-COX-2 polyclonal antibody, No. 4842S; β-actin = 1:1000 dilution, anti-β-actin polyclonal antibody, No. 4967S; Cell Signaling Technology, BCG, Mass, USA) in TBS-T buffer containing 5% BSA. The blots were treated with horseradish peroxidase-conjugated secondary antibody (1:2000 dilution goat anti-rabbit-IgG-HRP; Cell Signaling Technology, BCG, Mass, USA) in TBS-T buffer containing 5% BSA for 1 h, and an immune complex was detected using the ECL detection kit.

### 3.11. Statistical Analysis

IBM SPSS Statistics software v.19 was employed for data elaboration and statistical analysis using a level of significance set at 95%. One-way analysis of variance (ANOVA) together with response surface experiments were employed to group extracts, based on statistically significant differences; different letters suggest significant differences (*p* < 0.05).

## 4. Conclusions

In the current study, a group of single-factor experiments coupled with RSM modeling were utilized to optimize the extraction conditions for phlorotannins from *U. pinnatifida* sporophyll. The optimal conditions obtained are: heating temperature of 170 °C, heating time of 5.2 h, and an ethanol concentration of 52% with a yield of 10.7 ± 0.2 mg GAE/g DW. The PEUPS exhibited strong antioxidant and anti-inflammatory activities in vitro. These findings suggest that there is a good potential for the utilization of PEUPS in the design and development of novel functional foods and food supplements.

## Figures and Tables

**Table 1:** Codes and levels of factors for response surface method (RSM) experiments.

Factor	Level		
	−1	0	1
Temperature (°C)	100	150	200
Ethanol concentration (%)	25	50	75
Time (h)	1	4	7

**Table 2:** Experimental design and results for optimization of extraction parameters by RSM.

No.	Temperature	Ethanol Concentration	Time	TPC (mg GAE/g DW)
1	0	1	−1	1.7
2	−1	−1	0	1.6
3	0	−1	1	8.9
4	0	0	0	10.3
5	1	1	0	6.7
6	−1	0	1	2.6
7	1	0	1	6.9
8	0	0	0	9.5
9	0	1	1	10.3
10	1	−1	0	6.6
11	0	0	0	10.3
12	0	−1	−1	1.9
13	−1	0	−1	0.9
14	1	0	−1	10.4
15	−1	1	0	1.2
16	0	0	0	10.2
17	0	0	0	10.1

**Table 3:** Analysis of variance for the fitted regression equation.

Source	Sum of Squares	df	Mean Square	F Value	*p*-Value Prob > F	Significance Level
Model	205.06	9	22.78	4.03	0.0399	*
A-Temperature	73.80	1	73.80	13.04	0.0086	**
B-Ethanol concentration	0.16	1	0.16	0.028	0.8715	
C-Time	24.36	1	24.36	4.31	0.0767	
AB	0.068	1	0.068	0.012	0.9159	
AC	6.81	1	6.81	1.20	0.3089	
BC	0.67	1	0.67	0.12	0.7403	
AA	45.65	1	45.65	8.07	0.0250	*
BB	32.95	1	32.95	5.82	0.0466	*
CC	11.07	1	11.07	1.96	0.2046	
Residual	39.61	7	5.66			
Lack of Fit	39.20	3	13.07	127.14	0.062	
Pure Error	0.41	4	0.10			
Cor Total	244.67	16				

* *p* < 0.05; ** *p* < 0.01.

**Table 4:** Predicted and experimental values of PEUPS.

Reaction Condition	TPC of PEUPS (mg GAE/g DW)	
	Observed	Predicted
A = 169.2 °C, B = 52%, C = 5.2 h	-	11.0
A = 170 °C, B = 52%, C = 5.2 h	10.7	-

## Supplementary Materials

The following are available online at https://www.mdpi.com/1660-3397/17/8/434/s1, Figure S1: Effects of PEUPS on cell viability in RAW 264.7 cells, Figure S2. Nine polyphenolic compounds in extracted PEUPS based on the optimal condition.

## Abbreviations

PEUPS	Phlorotannins extracts from *U. pinnatifida* sporophyll
RSM	Response surface method
EGCG	Epigallocatechin gallate
ROS	Reactive oxygen species
iNOS	Inducible nitricoxide synthase
COX-2	Cyclooxygenase-2
TPC	Total phlorotannin content
GAE	Gallic acid equivalent
TEAC	Trolox equivalents antioxidant capacity assay
HBSS	Hank’s Balanced Salt Solution
NO	Nitric oxide
TBST	Tris-buffered saline Tween-20
GAE/g DW	Gallic acid equivalent/g Dry weight
