# Supplementary material for: Phlorotannins from Undaria pinnatifida Sporophyll: Extraction, Antioxidant, and Anti-Inflammatory Activities
Source: Mar Drugs. 2019 Jul 24;17(8):434. doi: 10.3390/md17080434 (PMC6723497; doi:10.3390/md17080434)
Supplement: Supplementary file 1 [file marinedrugs-17-00434-s001.pdf]

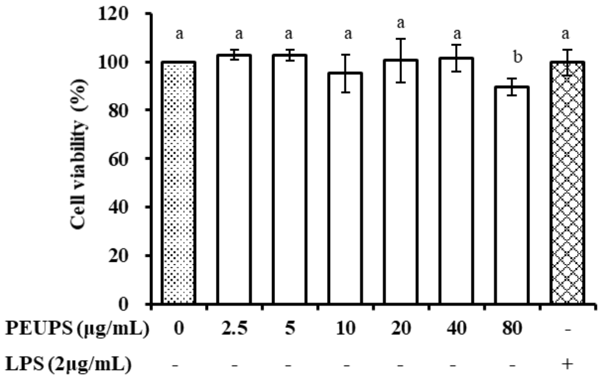

Figure S1. Effects of PEUPS on cell viability in RAW 264.7 cells. Cells (4×105 cells/mL) were incubated for 24 h with the indicated concentrations (2.5, 5, 10, 20, 40, 80 µg/mL) of PEUPS and 2 µg/mL of LPS. The cell viability assay was performed using MTT assay. Values are means ± SE (n = 3). Bars not sharing a common letter are significantly different (p < 0.05) from each other.

(a)

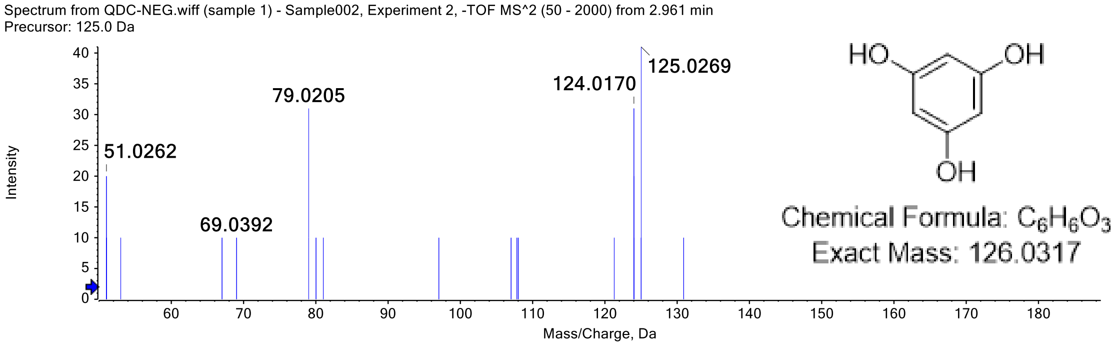
Spectrum from QDC-NEG.wiff (sample 1) - Sample002, Experiment 2, -TOF MS^2 (50 - 2000) from 2.961 min
Precursor: 125.0 Da
Intensity
Mass/Charge, Da
51.0262
69.0392
79.0205
124.0170
125.0269
HO
OH
OH
Chemical Formula: C6H6O3
Exact Mass: 126.0317

(b)

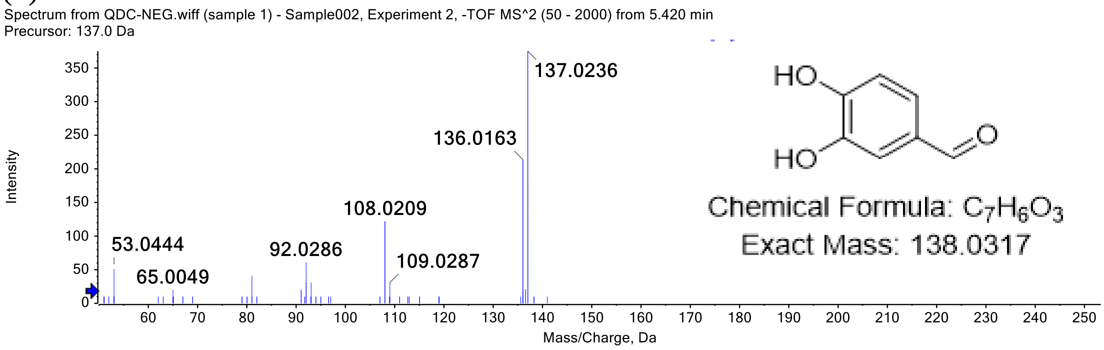
Spectrum from QDC-NEG.wiff (sample 1) - Sample002, Experiment 2, -TOF MS^2 (50 - 2000) from 5.420 min
Precursor: 137.0 Da
Intensity
Mass/Charge, Da
53.0444
65.0049
92.0286
108.0209
109.0287
136.0163
137.0236
HO
HO
O
Chemical Formula: C7H6O3
Exact Mass: 138.0317

(c)

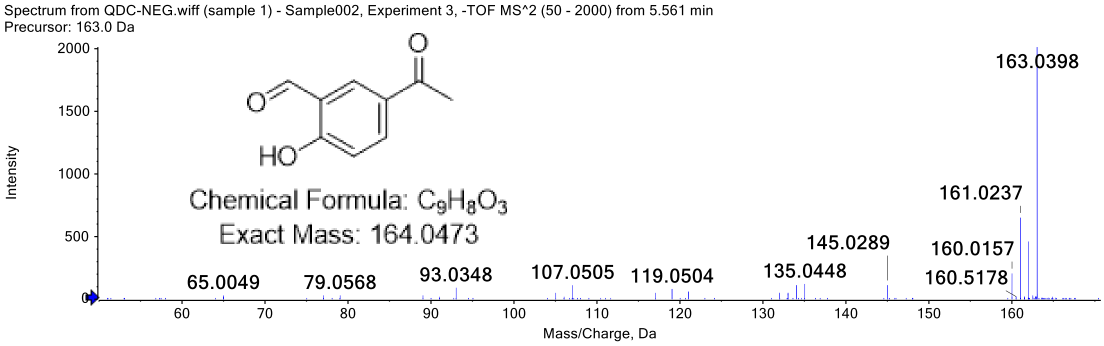
Spectrum from QDC-NEG.wiff (sample 1) - Sample002, Experiment 3, -TOF MS^2 (50 - 2000) from 5.561 min
Precursor: 163.0 Da
Intensity
Mass/Charge, Da
O
O
HO
Chemical Formula: C9H8O3
Exact Mass: 164.0473
65.0049
79.0568
93.0348
107.0505
119.0504
135.0448
145.0289
160.0157
160.5178
161.0237
163.0398

(d)

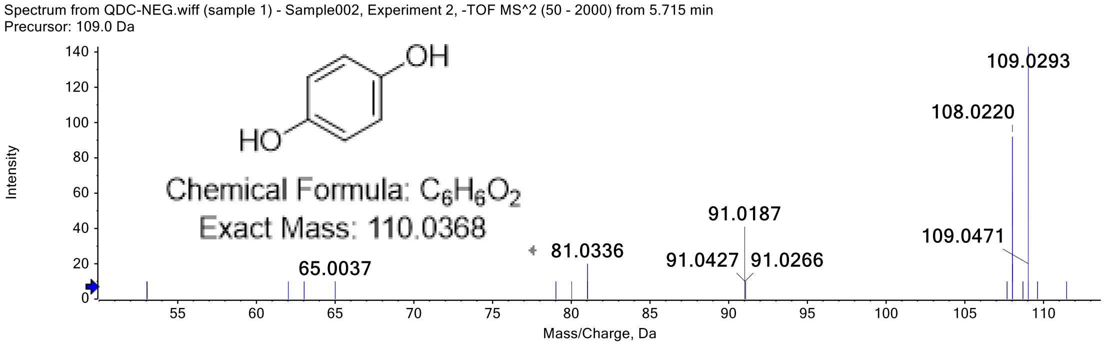
Spectrum from QDC-NEG.wiff (sample 1) - Sample002, Experiment 2, -TOF MS^2 (50 - 2000) from 5.715 min
Precursor: 109.0 Da
Intensity
Mass/Charge, Da
OH
HO
Chemical Formula: C6H6O2
Exact Mass: 110.0368
65.0037
81.0336
91.0187
91.0427
91.0266
108.0220
109.0471
109.0293

(e)

Spectrum from QDC-NEG.wiff (sample 1) - Sample002, Experiment 3, -TOF MS^2 (50 - 2000) from 6.594 min
Precursor: 151.0 Da

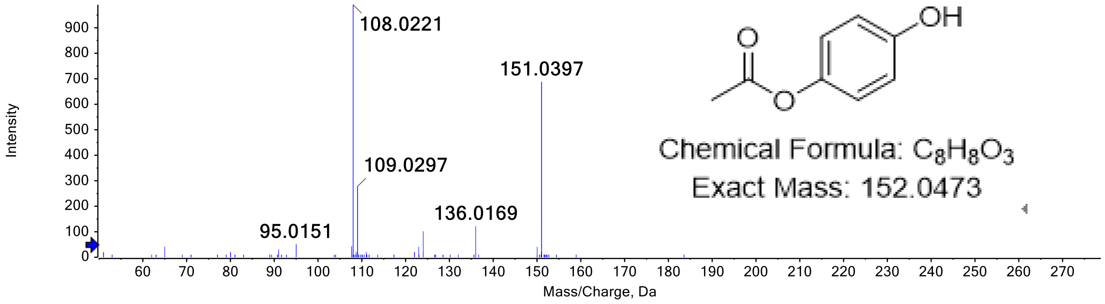

(f)

Spectrum from QDC-NEG.wiff (sample 1) - Sample002, Experiment 2, -TOF MS^2 (50 - 2000) from 7.914 min
Precursor: 121.0 Da

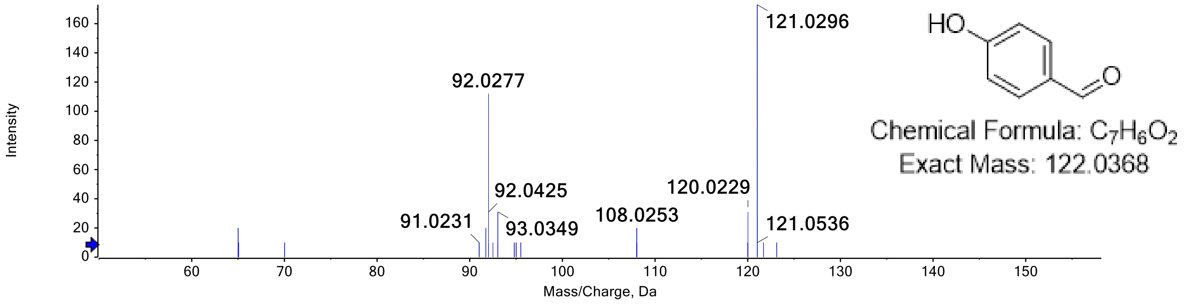

(g)

Spectrum from QDC-NEG.wiff (sample 1) - Sample002, Experiment 2, -TOF MS^2 (50 - 2000) from 8.852 min
Precursor: 151.0 Da

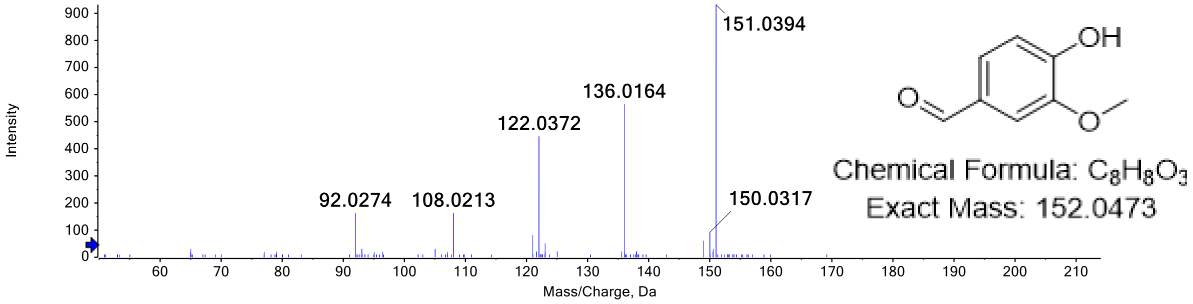

(h)

Spectrum from QDC-NEG.wiff (sample 1) - Sample002, Experiment 2, -TOF MS^2 (50 - 2000) from 10.070 min
Precursor: 177.1 Da

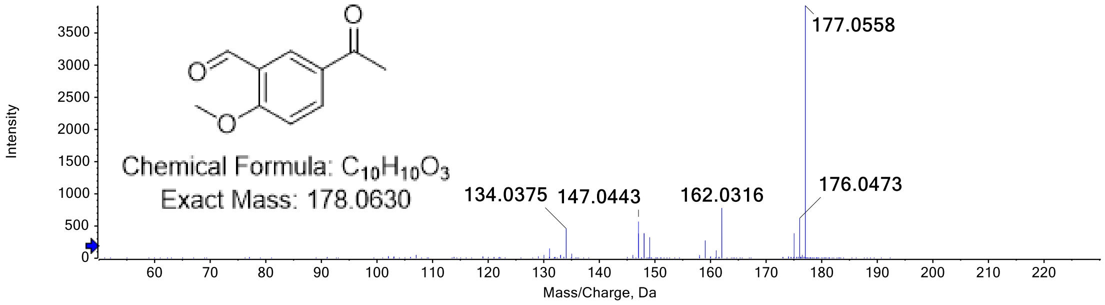

(i)

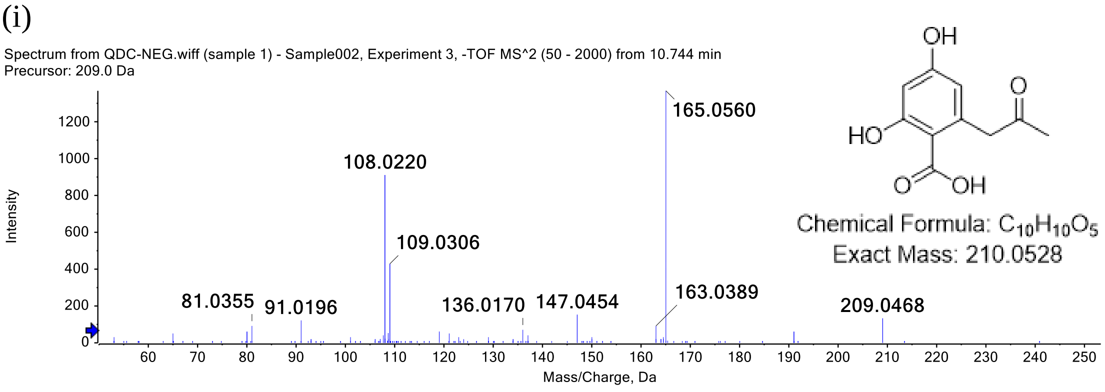

Figure S2. Nine polyphenolic compounds in extracted PEUPS based on the optimal condition. (a) 3,5-dihydroxyphenol; (b) 3,4-dihydroxybenzaldehyde; (c) 5-acetoxy-2-hydroxybenzaldehyde; (d) hydroquinone; (e) hydroquinone monoacetate; (f) hydroquinone monoacetate; (g) C8H8O3; (h) 1-(3-methoxy-4-hydroxyphenyl) ethenone; (i) 4-methoxy-phenol
